# Is reflection like soap? a critical narrative umbrella review of approaches to reflection in medical education research

**DOI:** 10.1007/s10459-021-10082-7

**Published:** 2021-11-12

**Authors:** Sven P. C. Schaepkens, M. Veen, A. de la Croix

**Affiliations:** 1 Department of General Practice, Erasmus University Medical Center, Postbus 2040, 3000 CA Rotterdam, The Netherlands; 2 Faculty of Medicine, Vrije Universiteit Amsterdam, Postbus 7057, 1007 MB Amsterdam, The Netherlands

**Keywords:** Critical narrative umbrella review, Philosophy, Reflection, Technicist and dynamic, Theory and practice

## Abstract

Reflection is a complex concept in medical education research. No consensus exists on what reflection exactly entails; thus far, cross-comparing empirical findings has not resulted in definite evidence on how to foster reflection. The concept is as slippery as soap. This leaves the research field with the question, ‘how can research approach the conceptual indeterminacy of reflection to produce knowledge?’. The authors conducted a critical narrative umbrella review of research on reflection in medical education. Forty-seven review studies on reflection research from 2000 onwards were reviewed. The authors used the foundational literature on reflection from Dewey and Schön as an analytical lens to identify and critically juxtapose common approaches in reflection research that tackle the conceptual complexity. Research on reflection must deal with the paradox that every conceptualization of reflection is either too sharp or too broad because it is entrenched in practice. The key to conceptualizing reflection lies in its use and purpose, which can be provided by in situ research of reflective practices.

## Introduction

The concept of reflection entered medical education based on new insights about what matters for becoming a competent professional (Sandars, 2009). Many undergraduate and specialty medical education programs include reflection to help medical trainees develop into competent professionals. Additionally, various theories about learning integrate reflection in their models, of which Kolb’s experiential learning is a prominent example (Caty et al., 2015; Coffield et al., 2004; Roessger, 2014). The main purpose of research on reflection in medical education is to support practice and its practitioners. Nonetheless, there is tension between theory and practice (van Enk & Regehr, 2018).

The reflective path towards professionalism is slippery like soap. Reflection is a complex phenomenon that lacks a theoretically unified concept. Empirical research on reflection shows a wide variety of methodological approaches (Fragkos, 2016; Mann et al., 2007). Some suggest that this is “due to the complexity of reflection itself, lack of consensus, and variability in educators’ understanding of the reflective process” (Uygur et al., 2019, p. 13). The multitude of definitions and models of reflection that currently exist contribute “to the accrual of multiple meanings of reflection” (Nguyen et al., 2014, p. 1184; Chaffey et al., 2012; Koole et al., 2011; Marshall, 2019; Roessger, 2014).

The underlying scientific problem can be captured in an analogy. If we ask adults to define soap, they might reply that soap consists of oil, lye, water and fragrance. If we ask a child the same question, she could say that it is a slippery thing that cleans dirty hands. A third reply might be poetic, like Francis Ponge’s *Soap*, that it behaves like a frog and a fish (Ponge, 2015). A chemist replies with C17H35COO- plus Na + or K + (Brenntag, 2021). Which of these descriptions captures the essence of soap? Is the chemist’s definition more fundamental than the child’s? Or should we combine them all? In short, what formulation unifies our understanding of soap?

Medical education research often strives for the scientific ideal of producing theory that can predict accurately and has ‘conceptual elegance’ (van Enk & Regehr, 2018). However, if reflection is a complex practice and behaves like soap, it might support a wide range of descriptions. Its very nature might resist the ideal of scientific rigor that equates to predictability and conceptual elegance (Heidegger, 2002). When a concept like reflection is important for medical education, it does not automatically follow that we can scientifically measure or assess it (Veen & Cianciolo, 2020). What does this imply for research on reflection in medical education?

A skeptic could argue that researching reflection requires modesty because it is so fundamental to being human. The concept is “just too big, that is, too general and vague, for effective, real world application” (Cornford, 2002, p. 226). Contrary to such skeptics, we argue that medical education research on reflection is sensible, provided we understand how different ways of operationalizing the concept affect the research. This requires us to take a step back, and clarify how medical education research can do justice to reflective practice without losing its conceptual and methodological integrity.

In this study, we conduct a philosophical analysis of the scholarly debate on reflection, which is challenged by its heterogeneous understanding of the concept of reflection itself. We aim to provide suggestions on how future conceptual development can take shape (Grant & Booth, 2009). Therefore, we address the questions, ‘what are the possible scientific approaches to conceptualizing and operationalizing reflection, and how do these influence knowledge production on reflection in practice?’.

## Methods

### Study design

We conducted a critical narrative umbrella review of research on reflection (Ng et al., 2015). This was a review of reviews, and included an analysis to ‘take stock’ and evaluate the previous body of work (Grant & Booth, 2009). We opted for this non-systematic review since many systematic reviews have already been performed without resulting in conceptual consensus.

### Data collection

We analyzed reviews of research on reflection in the broadest sense, ranging from systematic to narrative reviews in English. SS conducted a search with the search string (‘REFLECTION’ OR ‘REFLEXIVE’) AND (‘REVIEW’), and (‘REFLECTION’ OR ‘REFLEXIVE’) AND (‘SYSTEMATIC REVIEW’ OR ‘LITERATURE REVIEW’ OR ‘NARRATIVE REVIEW’ OR ‘CRITICAL REVIEW’) from 2000 until 6 July 2020 in the following databases: EMBASE, PubMed, Scopus, and Web of Science. Reviews eligible for inclusion in this study either had to report empirical research on (certain aspects of) reflection in medical education and/or medical health professions, or alternatively, regardless of their disciplinary focus, provide a substantial theoretical discussion on the concept of reflection (Brown et al., 2019). We included reviews from fields other than medical education, such as the teacher education field, when they dealt with cross-disciplinary theoretical discussions on reflection. We excluded studies that solely dealt with empirical research on reflection in non-medical education contexts. These in- and exclusion criteria resulted in 47 articles that we analyzed (cf. references with *).

### Analysis

Often, the purpose of reviews is to pool and assess empirical data, or synthesize literature about a phenomenon in a model or theoretical framework. For our study, however, the assessment of research and synthesized frameworks in the reviews *themselves* were the object of philosophical analysis. Philosophy is a broad field, but we understand it as “an academic discipline specialized in analyzing and understanding the wider processes of the constructing of theories, questioning their hidden background premises, and revealing and examining the values affecting (…) human practices” (Ruitenberg, 2009, p. 325; Holma, 2009). Philosophy can help identify different theoretical orientations on reflection, and “act as a broker or negotiator” between them, because it is not immediately obvious which approach is appropriate (Veen & Cianciolo, 2020, p. 5).

We analyzed literature reviews on reflection research by contrasting the foundational philosophical work that underpins the reviews with their assessments of reflection research. In this study, we do not describe *what* the trends are, but analyze *how* research produces scientific knowledge on reflection in alignment with reflection’s theoretical underpinnings. Using the foundational literature in this study functions like a ‘subjectivist lens’ (Varpio et al., 2020). The lens helps us recognize the field’s main tendencies that are valuable for analytical juxtaposition to highlight conceptual problems (Biesta, 2009; Davis, 2009).

## Results

We now present the results of our analysis by first describing the two distinct approaches to reflection research. We then describe each approach in more detail, discuss how they relate to each other based on our analytical lens, and provide further recommendations.

### Two approaches

We distinguished two main approaches to studying the ‘fuzzy concept’ of reflection. The first approach aims to dissolve conceptual ambiguity (Nguyen et al., 2014). This approach portrays a pressing need for unified conceptual ground between theories and empirical studies on reflection (Koole et al., 2011; Kuiper & Pesut, 2004; Kurt, 2018; Marshall, 2019; Nguyen et al., 2014). The second approach, in contrast, does not aim at dissolving ambiguity with an all-encompassing concept of reflection; instead, it incorporates the heterogeneous understanding of reflection as a vital quality in research on reflection (Mantzoukas, 2008; Ng, 2012; Ng et al., 2015; Norrie et al., 2012; Platt, 2014; van Beveren et al., 2018).

The reviewed studies mention various scholars such as Habermas or Kolb as influential in this field, but a clear majority refer to the work of Dewey and/or Schön as foundational (Erlandson & Beach, 2008; Fendler, 2003; Fragkos, 2016; Koole et al., 2011; Miraglia, 2015; Ng et al., 2015; Richard et al., 2019; van Beveren et al., 2018). Dewey’s and Schön’s work underpin both approaches to studying reflection. Although Schön responds critically to Dewey, there is a distinct commonality between them. With their study of reflection, they rethink the relation between theory and practice, and grant practice center stage (Erlandson & Beach, 2008; Farrell, 2012). Some studies question how far Dewey and Schön succeed; nevertheless, their work on reflection marks a pivotal point in this discussion (Hébert, 2015; Newman, 1999). Our ‘move’ in the philosophical analysis was, therefore, to adopt Dewey’s and Schön’s ideas as our analytical lens. From their work, we drew the qualities of a *technicist* and a *dynamic* rationale, that tie in with the theory–practice debate. We applied these two rationales to the contemporary scientific knowledge production on reflection.

Our use of the term *technicist* stems from what Schön describes as ‘Technical Rationality’ (Kinsella, 2007; Schön, 1983). *Technicist* indicates that professional problems are clearly demarcated and then solved by rigorously applying (scientific) theory and techniques in practice. ‘Applying’ implies a normative hierarchy, wherein “general principles occupy the highest level and concrete problem solving the lowest” (Schön, 1983, p. 24; Garrison et al., 2012). Theory and practice are mostly perceived as separated from each other, wherein theory is the systematic instrument bringing order to reality’s chaos (Kinsella, 2007). Both Dewey and Schön challenge this dualism between rationality and practice (Garrison et al., 2012).

Our use of the term *dynamic* originates with the description that Dewey and Schön give of experience and practice. They are dynamic, in the sense that problems in practice are not clear-cut but interconnected. Practice is messy, turbulent, filled with normative tensions, disorder, and conflict which make practice indeterminate (Kinsella, 2007; Ng et al., 2020; Schön, 1983). Furthermore, “rationality emerges over time in experience” (Garrison et al., 2012, p. 42). Whereas logic is unchanging and indifferent to context, “thinking is a process” that continuously changes because thinking always “has reference to some context” (Dewey, 1933, p. 72). In the next section, we will discuss the two approaches in detail.

### The technicist approach to reflection

Scholarship across the field of medical education research emphasizes that reflection is a complex phenomenon. Reflection is not seen as monolithic and one-dimensional like a switch that trainees can turn on or off. Instead, reflection moves along a continuum that happens over time (Koole et al., 2011; Marshall, 2019; Nguyen et al., 2014; Uygur et al., 2019). Such interpretations align well with a dynamic interpretation of reflection because it acknowledges that reflective practice has no clear-cut order. Nonetheless, upon closer scrutiny, studies that claim reflection is complex can still uphold technicist presuppositions that lead to particular technicist research problems and methodological solutions.

Many reviews conclude that the wide range of methodologies used to study reflection is problematic. They claim that effects of reflection are difficult to quantify and measure because multiple frameworks are in use. Cross-comparing research outcomes to harvest strong evidence yields limited results (Anderson et al., 2019; Bjerkvik & Hilli, 2019; Buckley et al., 2009; Chen & Forbes, 2014; Choperena et al., 2019; Contreras et al., 2020; Mann et al., 2007; McGillivray et al, 2015; Roessger, 2014; Uygur et al., 2019; Winkel et al., 2017). Ongoing theoretical disagreement over the definition of reflection is perceived as detrimental to validity and evidence-based practice (Marshall, 2019). The lack of consensus is seen as perpetuating “considerable uncertainty about how to best foster [reflection]” (Koole et al., 2011, p. 7), or prevents us from knowing if reflection is effective (Roessger, 2014; Winkel et al., 2017). Therefore, “a more defined construct of reflection, with clear outcomes, could lead to the development of benchmarks useful in tracking student progress and as research outcome measures” (Chaffey et al., 2012, p. 202).

To address the methodological challenges, studies with a technicist orientation synthesize common traits of reflection in models and definitions. They aim for “a comprehensive yet precise understanding of reflection” to accrue consensus that validates the concept (Marshall, 2019, p. 397; Koole et al., 2011; Kuiper & Pesut, 2004; Nguyen et al., 2014). Research tends to define reflection in generic terms to make the concept self-contained and untied to extrinsic elements for easier operationalization. “Reflection thus remains universally applicable and understandable independent of context” (Nguyen et al., 2014, p. 1185). Various reviews welcome such pursuits of systematized, de-contextualized models that clearly demarcate boundaries to mitigate conceptual ambivalence (Uygur et al., 2019).

Reviews with a technicist orientation stress the educational need for conceptual clarity and homogeneity. First, it caters to curriculum leaders who seek practical guidelines and validated assessment and feedback instruments. Such tools show whether trainees obtained the required skills, knowledge and attitudes and allow for more focused, structured and effective feedback (Chaffey et al., 2012; Koole et al., 2011; Nguyen et al., 2014). Moreover, standardized models and instruments help educators assess reflection outcomes uniformly and thus fairly. Second, this approach helps trainees gain a procedural understanding of reflection as a process that goes through certain phases with different dimensions that can be mastered.

The aforementioned benefits to practice are significant. However, up to this point, the field itself acknowledges its systematic failure to accurately measure effects or validly cross-compare studies to produce generalizable evidence. From the technical perspective, the solution lies with conceptual homogeneity and consensus that allows scientific standardization. Thus, the problem of reflection is complex, but in time its complexity can be instrumentally tamed because “there is nothing as practical as good theory” (Nguyen et al., 2014, p. 1187). Despite setbacks, the truth of reflection is ultimately perceived as testable in reference to the facts based on methodological rigor, accurate models and strict definitions (Schön, 1983).

### The dynamic approach to reflection

Like technicist oriented studies, those that adopt a dynamic approach to studying reflection also emphasize its complexity. Contrary to techniscist studies, however, they place theoretical emphasis on the messy nature of practice, and prioritize this over conceptual consensus and a universal definition. “Practice is characterized by uncertainty, instability, uniqueness, and value conflict, and (…) this is where the important questions of practice are negotiated” (Kinsella, 2009, p. 6; Mantzoukas, 2008; Ng et al., 2015). Reflective practice is thus “not as a fixed trait, but, rather, a dynamic state arising out of personal experience and sources of knowledge” (Ng et al., 2020, p. 6). This view supports the argument that reflection is not only complex, but more importantly, open. “Different practices and forms of thinking are considered reflective and the teaching of reflection is attributed to a broad diversity of educational values and purposes” (van Beveren et al., 2018, p. 7; Beauchamp, 2015). Developing a nuanced view on reflective practice rejects “a one-sized solution for facilitating ‘real’ (…) reflection” (Platt, 2014, p. 50).

Divergent views on reflection are empirically identifiable across the field. In their review, Norrie et al. (2012) conclude that there are significant variations in understanding reflection between healthcare professions. “In the medical context, the focus is on improving professional practice (…). In contrast, in other professions [e.g. nursing], reflective practice is approached more as a way of asserting each group’s autonomous professional identity” (Norrie et al., 2012, p. 573). Research in the medical field tends to favor a realist and pragmatic approach that is outcome oriented, with an emphasis on assessment and skill acquisition. Nursing adopts a more constructivist approach that is value-oriented. This varied production of reflection literature “is related to the history and traditions within the professions as well as to evolving national debates and policy imperatives” (Norrie et al., 2012, p. 574). Thus, the point of dynamic approaches is not synthesizing widely adopted reflective theories into one overarching, objective concept; rather, the meaning of reflection is entrenched in professional values stemming from practice with its own traditions and history. The concept of reflection is not universal, but open and socially contingent (Beauchamp, 2015).

Dynamic approaches suggest a revaluation of generalizable evidence. For instance, Mantzoukas (Mantzoukas, 2008) argues that the gold standard in Evidence Based Practice for reflection is not always a Randomized Control Trial. On the contrary, nurses “come to realize that reflection can provide not only valid evidences for practice, but possibly [allows them to be] positioned in a better place to provide more practical, useful and effective evidences” (Mantzoukas, 2008, p. 221). Rolfe argues that “what is required is not a science of large numbers, but a science of the unique. (…) [N]ursing science requires theories about individual persons,” that can also come from individual practitioners (Rolfe, 2006, p. 40).

The central message of scholarship that adopts a dynamic view of reflection is that “the field must broaden its conceptualization and deepen its understanding of what reflection is, from what philosophical contexts it derives, and what its purposes in the current socio-political context of medical education *can* be” (Ng et al., 2015, p. 469). Instead of diminishing reflection’s openness with precise models, scholarship should embrace openness that is infused by divergent practices. Furthermore, addressing context-specific socio-political dimensions incites debate, instigating “multiple ways of thinking about complex challenges in medical education” (Ng et al., 2015, p. 469).

## Discussion

### A paradox

Thus far, we have organized the research on reflection in a narrative, perceived through the lens of technicist and dynamic approaches. These approaches embody two different ways of tackling reflection’s complexity. A technicist approach to studying reflection has merit for practice, for instance by offering generalized and validated guidelines for education developers, trainees and practitioners. However, this merit is based on a consensus on the concept of reflection in order to subject it to solid empirical testing. From the dynamic perspective, this consensus still remains an ideal (Williams et al., 2019)–or rather, an impossibility. To further our understanding of both positions, we juxtapose the technicist and dynamic approaches.

We can see researchers of reflection struggle with a reciprocal tension between practice and theory, especially when research wants concepts to function anywhere, at any time and any place. From a technicist perspective, self-contained concepts that reduce reflection to its essence can be applied in any context. From a dynamic perspective, such reductionist definitions can end up in a ‘double bind’ (Ng et al., 2015): a self-contained concept that necessarily generalizes key components of the phenomenon to exclude alternatives, but in the process compromises the complexity which it wishes to convey (Kinsella, 2009). The disadvantage is that “many things that actually occur are debarred from use” (Sacks, 1985, p. 25). Conversely, reality rarely fully corresponds with abstracted prescriptions (Nofke & Brennan, 2005).

Generally, complex concepts show tensions between theory and practice. Tensions become tangible when research extrapolates the research object, like reflection, in a void as a self-contained concept. Borrowing from Wittgenstein’s work, concepts gain substance and significance in their situated use (Newman, 1999; Wittgenstein, 1958). For example, ‘soap’ gains concrete meaning within the context of washing your hands. To speak with the poet Ponge, soap is like no other stone found in nature. It gifts itself to you almost inexhaustibly after you marry it with water (Ponge, 2015). From a purely chemical context, soap is hydrophilic and hydrophobic; it can be ‘water-loving’ or ‘water-fearing’. In short, any phenomenon appears differently in distinct practices that show some overlap, but they are never completely identical. Likewise, each instance of reflection “resemble[s] others in many different ways, like the faces of people belonging to the same family,” but defining the vital essence between them is nigh impossible (Pears, 1970, p. 108; Wittgenstein, 1958). Meaning of complex concepts is in “the fine grain of events and processes” (Davis, 2009, p. 372).

To preserve the complexity and richness of reflection while trying to capture it in an all-encompassing concept provides research with a *paradox*. Each time we think of concepts, we deduce and reconstitute their meaning from their specific application in everyday use. Conversely, it is impossible to bring all varied uses to mind (Newman, 1999). A concept that needs to encompass all varied uses as much as possible will become too broad and will lose its power in the process (nearly everything can be called reflection). Simultaneously, the concept can become too narrow and precise, and cut away things that can also be seen as reflection. This paradox explains why scholars claim that reflection is notoriously difficult to define. Nonetheless, “without a context, the life of a concept is left without oxygen” (Nauta, 1984, p. 364; Boud & Walker, 1998; Flyvbjerg, 2006). This raises the question: why can the paradox manifest itself so prevalently in research on reflection?

### Reflection as a thick concept

We suggest that the paradox of formulating concepts too narrowly and too broadly, can be unpacked by thinking of reflection as a *thick concept* (Kirchin, 2013; Kroes & Meijers, 2016). On the one hand, thin concepts have an evaluative dimension. The barest examples are the words ‘pro’ and ‘con’, that indicate the simplest form of favoring or disfavoring something. On the other hand, thick concepts also have this evaluative function, but in addition they tell us something about a phenomenon. For instance, something *is* reflection when it has features of < a, b, c > , and *is not* reflection when < d, and e > . “The key problem here is whether we can be certain that we will ever capture all of [the phenomenon’s] instances” (Kirchin, 2013, p. 9). The thicker the concept the more local it becomes. ‘Pro’ and ‘con’ can be used almost universally, and can be transported beyond a distinct web of practices and meanings (Harcourt & Thomas, 2013, p. 24). Thicker concepts, however, are inherently bound to practice that thrives on some form of agreement in action among its participants (Medina, 2004). The crux of the matter is that socially complex phenomena are under constant *interpretation* because they involve countless interrelated elements; their meaning “cannot be simply ‘read off’ by direct observation” (Davis, 2017, p. 293).

We should not forget that reflection in medical education is not there for its own sake. There is always a purpose or point to reflection, but reflective practice is never fully stable because it needs (normative) interpretation. The point hinges on traditions and evolves from its socio-cultural history and ongoing debates (Norrie et al., 2012). For example, in the case of reflection in practice, there are broadly speaking two opposing points. On the one hand, reflection could imply *alignment* with new situations, and the point is ongoing socialization. This type of reflection could be, up to some degree, measured and assessed. ‘Effective and skilled reflection’ on the part of the trainee entails that potential gaps of knowledge are identified, for example, in reflective portfolios and subsequently addressed as learning goals. Successful identification of knowledge gaps indicates that reflection was effective. On the other hand, one could argue that reflection instigates *deviation* from institutional norms by becoming explicitly critical of current practice. Measuring and assessing critical reflection is nonsensical, because assessment safeguards the very institutional ideals that critical reflection is supposed to question (Hodges, 2015; Ng et al., 2020; Procee, 2006). Other points of contention are: should reflection be about emotions, or if it should be rational, debar from emoting (Birden & Usherwood, 2013; Nguyen et al., 2014; Wald, 2015), is reflection a solitary or interpersonal activity (Kotzee, 2012), can reflection lead to harmful rumination (Lengelle et al., 2016), and can we measure reflection (Aukes et al., 2007; de la Croix & Veen, 2018; Veen et al., 2020)? With each of these and other contested areas, the point of reflective practice is always at stake. Various points are incongruent and prevent a universal *description* of reflection from materializing. Thicker concepts place more demands on explaining the social situation they function in than thin concepts, while any concept equips the user with reasons for doing things (Harcourt & Thomas, 2013). Each conceptualization and application of reflection in practice is a temporary depiction of the normative debate in academia and educational institutes (Gu-Ze'ev et al., 2001; Norrie et al., 2012). This leaves research with the challenge: how can we study a debated concept like reflection?

### How can we study reflection?

Given the contested state of reflection, how should we study it? The key limitation of our approach is that we interpret the philosophical underpinnings as coherently and consistently as possible, but that no conclusive interpretation exists. Nonetheless, medical education often proffers to be an interdisciplinary field, but the reality is that most medical education research is still done from the perspective of medical research (Albert et al., 2020). Perhaps we can achieve a ‘multidisciplinary edge effect’ (Varpio & MacLeod, 2020) through fruitful dialogue between technicist and dynamic approaches.

Researchers who adopt a technicist approach should be aware that their outlook on science is more in alignment with the epistemic culture from medical research, and mostly methodologically deductive in nature (Varpio & MacLeod, 2020). This means that reflection is usually conceptualized upfront and tested in diverse circumstances. The value of this approach is in checking if the chosen variables appear as outcomes in specific practices by using datasets like questionnaires, portfolios, rubrics or (statistical) analyses of learning outcomes. In general, this approach accepts that only features of reflection in practice that are adopted in the initial conceptualization will necessarily appear in the outcomes–‘you will find what you formulate upfront’. Those features that have not been conceptualized upfront will likewise not appear in the results (Sacks, 1985; Uygur et al., 2019). The research is mostly *prescriptive*. First, research is prescriptive in a normative sense, because the conceptualization prescribes what should happen in practice (van Enk & Regehr, 2018). Simplified, if one were to conceptualize reflection as a purely ‘rational dissection of events,’ then emotional moments are not flagged as reflective. Second, and in line with Dewey, research is prescriptive in a methodological sense because all inquiry is concept-laden. Concepts direct observation and demarcate relevant from irrelevant information (Garrison et al., 2012). This need not be a problem, if we keep in mind that the selection of a (thick) concept is temporary and limited. Concepts are contingent and subject to continuous reconstruction. Claiming conceptual universality by aggregating all available theories will remain, in our view, idealistic.

Technical approaches aim for self-contained concepts in generic wording to facilitate operationalization, “unlike, for example, Schön’s model, which is not easy to grasp without lengthy exploration of his writing” (Nguyen et al., 2014, p. 1185; Koole et al., 2011). For example, Marshall’s concept of reflection, after synthesizing theories across different professional contexts, reads as follows: “Reflection is a careful examination and bringing together of ideas to create new insight through ongoing cycles of expression and re/evaluation” (Marshall, 2019, p. 411). This concept is theoretically ‘correct’ and helps research gain a first footing, but from a dynamic and Wittgensteinian perspective, it remains abstract, up to the point that it means very little. What do ‘careful examination,’ ‘bringing together ideas,’ ‘ongoing cycles of expression’ and ‘re/evaluation’ look like? How do practitioners go about accomplishing such feats? What do they do, say or remain silent on? The concept still needs to come alive by aligning it closely with practice.

From the dynamic approach, we take the value of (single) case studies (Flyvbjerg, 2006; Newman, 1999; Rolfe, 2002, 2006). Case studies give abstract concepts oxygen. From the dynamic perspective, case studies are not designed to mean the same thing to all people. The case study should be sufficiently rich with so many facets, mimicking practice itself, that “different readers may be attracted, or repelled, by different things in the case. Readers are not pointed down any one theoretical path or given the impression that truth might lie at the end” (Flyvbjerg, 2006, p. 238). The case studies reject “the certainty of any one meaning implied by the single term ‘reflective practice’” (Newman, 1999, p. 160). In the current research field, we see that researching reflective practice in situ to provide more dynamism to technicist abstractions is still underrepresented. Studies that examine reflection ‘as it occurs in practice,’ for instance with conversation analysis of reflection group sessions (van Braak et al., 2018; Veen & de la Croix, 2016), or phenomenological approaches (Rietmeijer et al., 2021) can provide additional dynamism.

## Conclusion

We asked what the possible scientific approaches to conceptualizing and operationalizing reflection are, and how these influence knowledge production on reflection in practice. Our analysis of medical educational literature on reflection showed that there are two main responses to reflection’s conceptual indeterminacy. The technicist and dynamic approaches both agree that reflection is complex, but technicists attempt to tame its complexity by seeking conceptual consensus on reflection. Consensus is beneficial to standardize and cross-compare research and understand how reflection is effective. Conversely, the dynamic approach embraces reflection’s conceptual openness, and emphasizes the importance of local practice. Practices are historically contingent and evolving, and thus reflection is theoretically variable. We interpreted reflection as a thick concept, and argued that research is bound to the paradox that any conceptualization is either too broad or too narrow. Contingent practice limits the reach of any theory, and universal formulations of reflection have strong limitations. Furthermore, the two approaches can be complementary; generalized technicist theory can come alive by providing (single) case study evidence of practice.

Finally, we come back to our poet friend, Ponge, who tried to describe soap, but there was so much to say that he returned it to its saucer. It appears as if Ponge is defeated. However, we feel that his poem’s last words on the matter are of key importance: “… it is necessary to return it to its saucer, to its strict appearance, its austere oval, its dry patience, and its power to serve again” (Ponge, 2015). Like an effort to understand and describe soap, the key to conceptualizing reflection lies in its use and purpose that inspires any description. Taking into account that a child’s definition of soap is different from that of a chemist is crucial. Each definition of soap or reflection will do different work. Combining all occurrences to find that definitive, universal definition will remain, for us, idealistic. However, reflection, like soap, *will serve again* if we return to its practice, which should not be underestimated.
